# Morphological Characterization and Effective Thermal Conductivity of Dual-Scale Reticulated Porous Structures

**DOI:** 10.3390/ma7117173

**Published:** 2014-10-28

**Authors:** Simon Ackermann, Jonathan R. Scheffe, Jonas Duss, Aldo Steinfeld

**Affiliations:** 1 Department of Mechanical and Process Engineering, ETH Zürich, Sonneggstrasse 3, 8092 Zürich, Switzerland; E-Mails: acsimon@ethz.ch (S.A.); jscheffe@ufl.edu (J.R.S.); jduss@student.ethz.ch (J.D.); 2 Solar Technology Laboratory, Paul Scherrer Institute, 5232 Villigen PSI, Switzerland

**Keywords:** tomography, solar, thermochemical, conductivity, reticulated porous ceramic (RPC)

## Abstract

Reticulated porous ceramic (RPC) made of ceria are promising structures used in solar thermochemical redox cycles for splitting CO_2_ and H_2_O. They feature dual-scale porosity with mm-size pores for effective radiative heat transfer during reduction and µm-size pores within its struts for enhanced kinetics during oxidation. In this work, the detailed 3D digital representation of the complex dual-scale RPC is obtained using synchrotron submicrometer tomography and X-ray microtomography. Total and open porosity, pore size distribution, mean pore diameter, and specific surface area are extracted from the computer tomography (CT) scans. The 3D digital geometry is then applied in direct pore level simulations (DPLS) of Fourier’s law within the solid and the fluid phases for the accurate determination of the effective thermal conductivity at each porosity scale and combined, and for fluid-to-solid thermal conductivity from 10^−5^ to 1. Results are compared to predictions by analytical models for structures with a wide range of porosities 0.09–0.9 in both the strut’s µm-scale and bulk’s mm-scale. The morphological properties and effective thermal conductivity determined in this work serve as an input to volume-averaged models for the design and optimization of solar chemical reactors.

## 1. Introduction

Foam-type reticulated porous ceramics (RPC) structures are applied in a broad range of physical processes requiring enhanced heat and mass transfer [1,2]. Applications include microelectronics cooling [3], soil dynamics [4,5], catalytic reactors [6], radiant burners [7], tissue engineering [3,8] and volumetric heat exchangers for the conversion of concentrated solar energy [9,10,11]. Of special interest of the latter application is the solar-driven thermochemical redox cycle for splitting CO_2_ and H_2_O [12,13,14,15,16], consisting of: (1) a high-temperature endothermic reduction, in which a metal oxide is thermally reduced and oxygen is evolved; and (2) a lower-temperature exothermic oxidation, in which the reduced oxide is re-oxidized with H_2_O and CO_2_ to form H_2_ and CO (syngas), and further processed to liquid hydrocarbon fuels. Ceria-based oxides have emerged as highly attractive redox materials because of the rapid oxygen transport in the bulk [14,15,16,17,18]. Various porous structures made of ceria have been investigated for enhanced reaction rates [18,19,20], including structures with submicron-sized interconnected pores, but these are problematic to retain because of partial sintering at elevated temperatures [19]. Furthermore, their high optical thickness inhibits penetration of concentrated solar radiation, resulting in non-uniform heating and temperature distributions [14]. Most recently, Furler* et al.* [15] presented a unique and morphologically stable RPC structure featuring dual-scale porosity: mm-size pores with struts containing micron-size pores. The mm-size pores enable volumetric absorption of concentrated solar radiation and effective heat transfer during the reduction step, while the micron-size pores within the struts offer increased specific surface area leading to enhanced reaction kinetics during the oxidation step.

Optimization of solar reactors for thermochemical redox cycles requires computational models of heat transfer and fluid dynamics coupled to the reaction kinetics [14,21]. Since resolving the solar reactor at the pore scale would require tremendous computational demand, volume-averaging theory is often applied for solving the mass, energy, and momentum conservation equations using effective heat and mass transport properties [22,23,24,25]. These can be determined accurately by direct pore-level simulations (DPLS) using the detailed 3D digital geometry of the structure obtained by computer tomography (CT) [26,27,28]. For example, the Monte Carlo ray-tracing method has been applied at the pore level for solving the radiative heat transfer equations and determining the effective extinction coefficient and scattering phase function [29], and the finite volume (FV) technique has been applied at the pore level for solving the Navier-Stokes equations and determining the effective thermal conductivity, permeability, and heat transfer coefficient [30,31].

**Figure 1:**
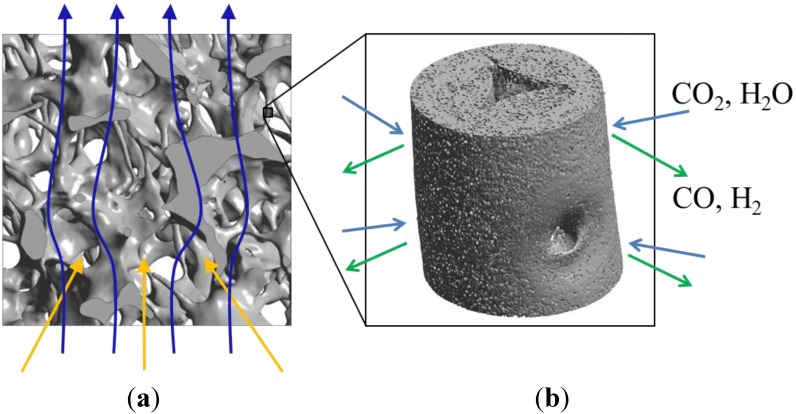
Ceria RPC with dual-scale porosity: mm-size pores for volumetric radiative absorption and effective heat transfer (**a**) during the reduction step, and struts containing micron-sized pores leading to increased specific surface area (**b**) for enhanced reaction kinetics during the oxidation step.

In this work we apply the tomography-based methodology to investigate RPC structures made of ceria with dual-scale porosity in the mm and µm scales. This structure is schematically depicted in Figure 1 [15]. The total and open porosity, pore size distribution, mean pore diameter and specific surface area are extracted from the CT-scans. The effective thermal conductivity is determined by DPLS for the RPC with non-porous struts and for the RPC with dual-scale porosity. We investigate the effect that the dual-scale porosity has on the morphological properties and on the conduction heat transfer across the RPC, and further compare the results to predictions by analytical models for structures with a wide range of porosities in both the strut’s µm-scale and bulk’s mm-scale.

## 2. Experimental

### 2.1. RPC Synthesis

The dual-scale RPC structure is manufactured using the Schwartzwalder foam replication method [32]. An organic foam template is coated with multiple slurry layers containing ceria particles and micron-sized carbon grains [15]. The carbon pore former content ranges from 10 to 50 vol%. After firing at high temperatures (>1800 K), the bearing carbon foam and grains are burned und the desired foam-type structure undergoes sintering.

### 2.2. Synchrotron Submicrometer Tomography

**Figure 2:**
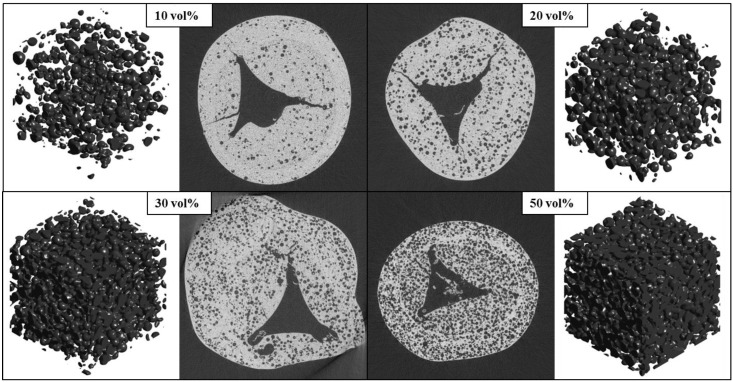
Synchrotron submicrometer computer tomograms of single RPC struts manufactured with various pore former concentrations (10, 20, 30, and 50 vol%) and their corresponding 3D digital reconstruction of the void space within isotropic porous strut region.

Strut samples are scanned using synchrotron submicrometer tomography with a voxel (3D pixel) size of *v*_s_ = 325 nm and a 0.832 × 0.832 × 0.702 mm^3^ field of view. The high-resolution CT is performed at the Swiss Light Source (SLS) of the Paul Scherrer Institute (PSI, Villigen, Switzerland) with the TOMCAT beamline for 40 keV photon energy, 400 µA beam current, a 100 µm thick aluminium filter, 40 µm thick copper filter, a 10 µm thick iron filter, 20× geometrical magnification, 1 s exposure time, 1001 projections. Figure 2 shows exemplary tomograms of strut samples manufactured with various concentrations of pore former ranging from 10 to 50 vol% and their corresponding 3D reconstructions of the pore space within isotropic strut regions. One tomogram contains 2560 × 2560 pixels. Numerous strut samples are scanned to verify reproducibility.

### 2.3. Micrometer Tomography

**Figure 3:**
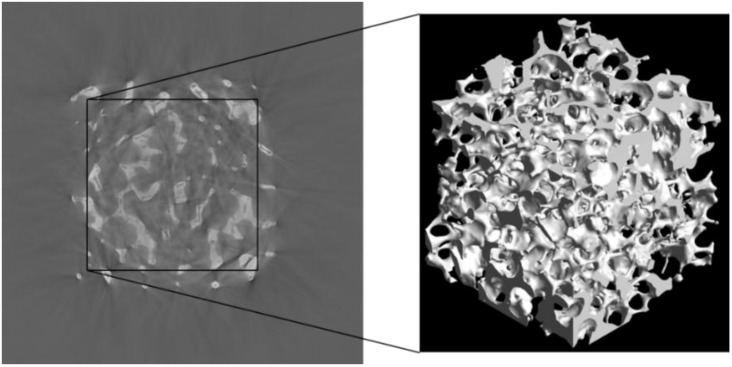
Computer tomogram of the RPC with mm-sized pores and 3D rendering of a cropped cubic sample.

A ceria RPC sample with 10 pores per inch (ppi) is scanned by micrometer tomography with a voxel size of *v*_s_ = 35.7 µm and a 36.56 × 36.56 × 36.56 mm^3^ field of view. The low-resolution CT is performed with an unfiltered polychromatic X-ray beam at the Swiss Federal Laboratories for Materials Science and Technology (EMPA, Dübedorf, Switzerland) for 150 keV photon energy, 45 µA beam current, 6.272 s exposure time and 721 projections. Figure 3 shows a tomogram and its corresponding 3D digital reconstruction of the scanned RPC sample. One tomogram contains 1024 × 1024 pixels.

## 3. Morphological Characterization

### 3.1. Porosity

For the analysis of the strut structures, isotropic regions within submicrometer tomograms are cropped with the size of 501 × 501 × 501 voxels (0.163 × 0.163 × 0.163 mm^3^). For the analysis of the RPC structures, micrometer tomograms are cropped to 500 × 500 × 500 voxels (17.85 × 17.85 × 17.85 mm^3^). The cropped 8 bit tomograms obtained from measurements are pre-processed with a 3D Gaussian blurring filter to remove unwanted image noise derived from the photon sensor. Histograms computed from 3D tomogram stacks show bimodal character representing two grey scale pixel classes, with the threshold found by Otsu’s method of intra-class variance minimization [33,34]. Finally, based on the threshold, each pixel is assigned to be either void or solid. Porosity, ε = *V*_f_/*V*, is defined as the ratio between the void space volume and the total cube volume. It is calculated by counting void and solid voxels of the 3D stack. The representative elementary volume (REV) defines the minimum volume containing a porous zone for which the continuum assumption is valid. It is determined from incrementally growing cubic subvolumes until their calculated porosities convergences within a certain band, ±γ. The conditions for the minimum edge length of the REV are [27]:

*l*_REV_ = min{*L* ≤ L* |ε − γ < ε(V_L*_) < ε + γ},  γ << 1 (1)

where V_L*_ is the sample subvolume and L* is the edge length of the sample subvolume. For the RPC with a porosity band of γ = 0.05, *l*_REV_ ≥ 6.6 mm, which leads to cube structures larger than 186 voxels edge length. For the µm-sized struts with a porosity band of γ = 0.05, *l*_REV_ ≥ 76.1 µm, which leads to cube structures larger than 235 voxels edge length.

Dual-scale porosity, ε_dual_, is calculated from strut-scale porosity, ε_strut_, and RPC-scale porosity, ε_RPC_, as:

ε_dual_ = ε_RPC_ + (1 − ε_RPC_) ∙ ε_strut_ (2)

ε_strut_ is linearly fitted to the pore former concentration, ϕ, as ε_strut_ = 0.008707 ϕ. RPC structures with varying strut thicknesses are generated by altering the original segmented tomography scans through a dilation process with 3D spherical elements of a certain diameter, *d*, with 
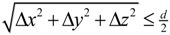
. Table 1 lists the dilation radius, digital porosity, and mean pore diameter of the original RPC reconstruction and of the digitally altered RPC for 3 increasing strut thicknesses, and the corresponding digital section cut and 3D rendering. As expected, porosity and mean pore diameter of the RPC decrease with increasing strut dilation because the thicker struts consume void space.

Of special interest is the connectivity of the µm-sized pores within the struts. The pore connectivity scales directly with the specific surface area reachable by reacting gases, and thus scales with the fuel production rates [15]. Open porosity, ε_open_, is defined as the pore space accessible from one of the 6 cube sample surfaces. An iterative routine starts searching from one cube side for connected neighbour void voxels in order to find all pores connected to this side. This reconstruction is performed to detect closed pores within the structure that account for the porosity but are not exposed to the gaseous reactants. Table 2 shows exemplary results of total and open porosity for strut samples with various pore former concentrations.

**Table 1:** Dilation radius, digital porosity, and mean pore diameter of the original RPC reconstruction and of the digitally altered RPC for increasing strut thickness, and the corresponding digital section cut and 3D rendering.

*r*_dil_ (voxel)	0	2	5	10
*r*_dil_ (mm)	0	0.071	0.179	0.357
ε_RPC_ (–)	0.823	0.756	0.644	0.459
*d*_mean_ (mm)	2.32	2.21	1.98	1.64
Digital section cut through 3D structure at* h* = 50%	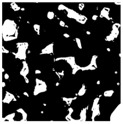	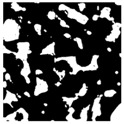	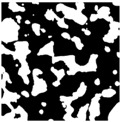	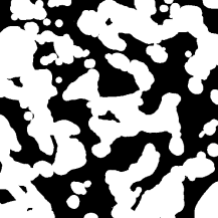
3D rendering of RPC with mm-sized pores	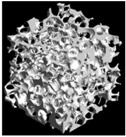	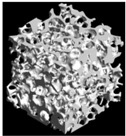	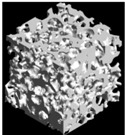	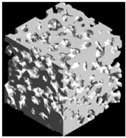

**Table 2:** Strut and open porosity, mean pore diameter, and the corresponding 3D rendering for strut samples manufactured with various pore former concentrations.

ϕ (vol%)	10	20	30	50
ε_strut_ (–)	0.1195	0.1797	0.2605	0.4436
ε_open_ (–)	0.0095	0.0102	0.2167	0.4423
*d*_mean_ (µm)	9.22	11.50	9.62	9.12
3D rendering of connected pore space	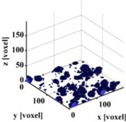	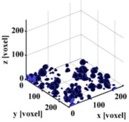	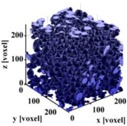	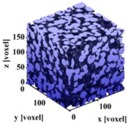

**Figure 4:**
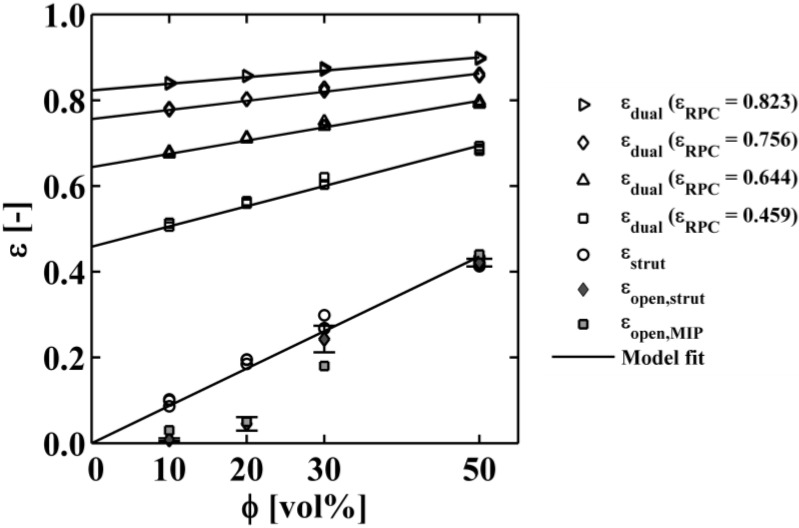
Total and open porosity as a function of the pore former concentration used to manufacture the µm-sized strut pores, total dual-scale porosity of RPC (original and digitally altered strut thickness).

Figure 4 shows all results of the porosities collected from selected 3D tomography reconstructions. ε_open_ is presented with error bars because 6 evaluations are obtained per sample (one from each side). For ϕ ≤ 20 vol%, there is no pore connectivity observed as seen graphically in Table 2. For ϕ ≥ 30 vol%, the majority of the pores are connected and the pore network passes through the entire cube sample. For 50 vol%, practically every pore is connected to the pore network since the open porosity and total porosity are almost the same ε_open_ ≈ ε_strut_. For high RPC porosity, e.g., ε_RPC_ = 0.825, the dual-scale porosity changes by less than 0.1 for ϕ = 50 vol% because only 17.5% of the volume is filled with µm-sized pores within the thin struts (see Figure 4). For lower RPC porosities, e.g., ε_RPC_ = 0.459, there is a stronger trend for increasing ϕ since there is more solid to be filled with µm-sized pores. The porosities of the model fit for ϕ = 0 vol% represent ε_RPC_ as listed in Table 1.

**Figure 5:**
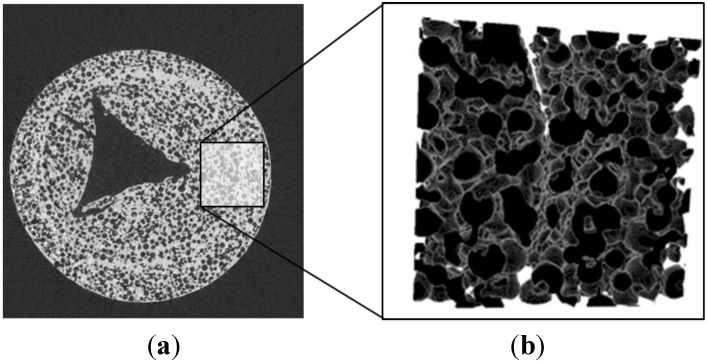
Poor pore connectivity across fabrication layer originating from 2-step coating. (**a**) tomogram of a strut sample manufacture with 50 vol% carbon grain pore former. (**b**) 3D rendering of the void phase with pronounced solid barrier across the two coating layers.

To investigate the transition from closed pores to interconnected pores in a systematic way, spheres with a small Gaussian size distribution of 2–3 voxels are randomly placed within the 3D volume. Pores of such artificially generated structures get connected between ε_strut_ = 0.2 and 0.3. Such connectivity behavior is consistent with empirical correlations of the effective gas diffusivity within porous carbon [35]. Figure 4 also shows the comparison between the numerically determined ε_open_ and the experimentally measured values by mercury intrusion porosimetry (MIP) [36,37], as reported by Furler* et al.* [15]. The agreement is reasonable well. For ϕ = 30 vol%, the experimentally measured ε_open_ is lower than the numerically determined one due to poor pore connectivity across coating layers, as seen in Figure 5. This anisotropic region results from the 2-step coating applied during the fabrication process, and it is not considered in the determination of morphological properties and effective thermal conductivity within isotropic regions.

### 3.2. Pore Size Distribution

Pore size distribution is determined by applying a morphology-altering algorithm to the digitally segmented 3D structure consisting of an inversion of the solid and void space, followed by erosion and dilation with successively increasing spherical elements of diameter *d* with 
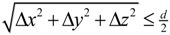
. In a last step, the algorithm inverts back the solid and void space. The cumulative pore size distribution 1 − *F*(*d*) is defined as the ratio of the opening-closing porosity, ε_oc_(*d*), and the original porosity [27]:

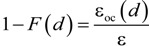
(3)


**Figure 6:**
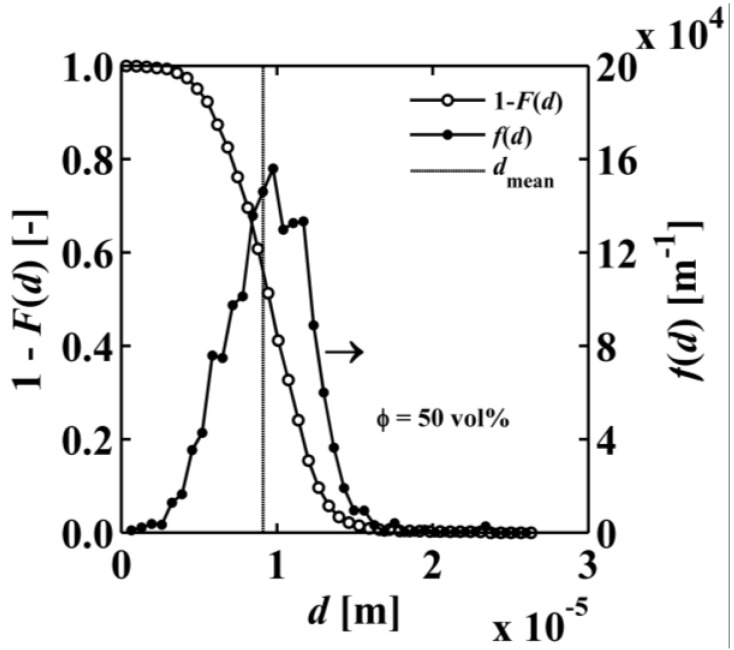
Cumulative pore size distribution (**left** *y*-axis) and pore size distribution (**right** *y*-axis) obtained by morphology operations with spherical structuring elements of diameter *d*.

The pore size distribution is then calculated as: *f*(*d*) = d*F*(*d*)/d*d*. Figure 6 shows the cumulative pore size distribution (left *y*-axis) and pore size distribution (right *y*-axis) as a function of *d*.·*f*(*d*) agrees qualitatively well with the values obtained by mercury intrusion porosimetry measurements. The mean pore diameter is then defined in such a way to split the area under the pore size distribution curve into two equal areas [27].

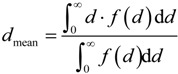
(4)


The mean pore diameter for porous strut samples with various concentrations of pore former in the range 10–50 vol% is listed in Table 2. As expected, *d*_mean_ remains independent of ϕ at around 10 µm because the same pore forming material is used to manufacture all samples.

### 3.3. Specific Surface Area

The specific surface area (SSA) is determined in three different ways: (1) using statistical two-point correlation function computed on the 3D segmented structures with an in-house Fortran code; (2) resampling of the phase interface area with a surface mesh-based algorithm using the open source software ImageJ (version 1.47v, Java 1.6.0_20 (64-bit)) [38] extended with the free BoneJ plugin (version 1.3.11) [39]; and (3) using the actual phase interface area of tetrahedral 3D meshes generated with an in-house Fortran code [40]. The in-house mesh generator covers the void and solid domain with tetrahedral elements for unstructured body-fitted grids and subsequently refines the elements at the phase boundary. These 3D meshes are later used to solve the steady-state energy conservation equation to determine the effective thermal conductivity. The two point correlation *s*_2_(*r*) is a statistical function that indicates the probability of two arbitrary points Ψ(*r*) and Ψ(**r** + *r***ŝ**) separated by the distance *r* to be in the void phase [41]:

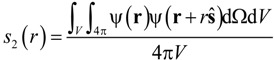
(5)
where Ω is the solid angle and *V* the cube volume. Porosity and specific surface area are then calculated using the following expressions [27,30]:

*s*_2_ (*r* = 0) = ε (6)

*s*_2_ (*r* → ∞) = ε^2^ (7)

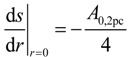
(8)


A code loops once through the entire structure (*x*-, *y*-, *z*-direction), counts each void voxel, and computes *s*_2_(*r* = 0) in a digitally exact manner. Additionally, the code counts for each void voxel the number of direct neighbour void voxels in all 6 directions (-*x*, *x*, -*y*, *y*, -*z*, *z*) and computes 6·*s*_2_(*r* = 1). The volumetric specific surface area is then calculated as:

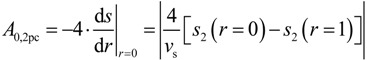
(9)


The specific surface area is presented in two different units: per total volume (fluid+solid phases), *A*_0_ [m^2^ · m^−3^], and per ceria mass, *ssa* [m^2^ · g^−1^]. The density of ceria is ρ_CeO_2__ = 7.22 g/cm^3^ [21,42]. Dual-scale *A*_0_ is calculated by multiplying *A*_0_ determined for the struts by the solid volume fraction of the RPC: *A*_0,dual_ = (1 − ε_RPC_) · *A*_0,strut_. *A*_0_ of RPC with non-porous struts is converted to *ssa* by: *ssa*_RPC_ = *A*_0,RPC_/[(1 − ε_RPC_) · ρ_CeO_2__]. Table 3 lists *ssa* of RPC with non-porous struts obtained from the original tomography scans and digitally dilated struts.

**Table 3:** *ssa* of RPC with non-porous struts obtained from tomography scans and digitally dilated struts.

*r*_dil_ (voxel)	0	2	5	10
*r*_dil_ (mm)	0	0.071	0.179	0.357
*ssa*_RPC,2pc_ (m^2^·g^−1^)	9.06 × 10^−4^	5.89 × 10^−4^	4.07 × 10^−4^	2.62 × 10^−4^
*ssa*_RPC.mesh_ (m^2^·g^−1^)	6.39 × 10^−4^	4.98 × 10^−4^	3.75 × 10^−4^	2.64 × 10^−4^

Because *ssa* is defined per unit mass, the strut *ssa* is necessarily equal to the *ssa* of the entire structure, including mm and µm sized pores. Thus, *A*_0,strut_ is used to calculate *ssa* of porous struts and dual-scale porous structures:

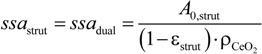
(10)


**Figure 7:**
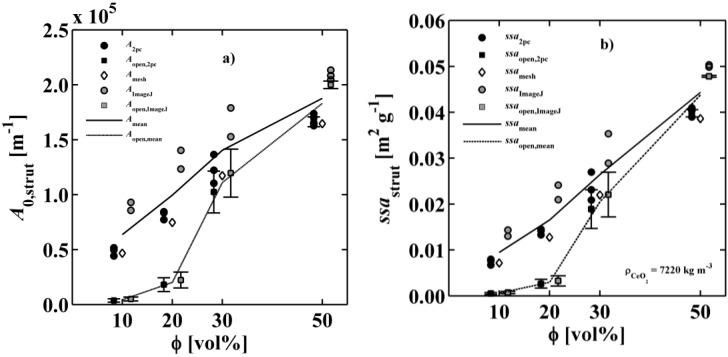
Total and open specific surface area, *ssa*, as a function of the pore former concentration used to manufacture the µm-sized strut pores: (**a**) volumetric specific surface area of µm-sized strut pores; and (**b**) specific surface area per ceria mass of porous struts.

*A*_0_ and *ssa* are plotted as a function of ϕ in Figure 7a,b, respectively. Of special interest is the open *ssa*, which is directly related to the surface area reachable by the reacting gases for conversion of CO_2_ and H_2_O to CO and H_2_. The open *ssa* is calculated for the struts without closed pores. Open *A*_0_ and *ssa* are presented with error bars (standard deviation) for the data evaluated. Surface areas calculated from two-point correlation and phase interface area of the 3D meshes (black and white symbols) lie within close proximity to one another, whereas those calculated using ImageJ (grey symbols) are higher. This is because the two-point correlation is based on a statistical model leading to smoothing effects and the 3D meshes actually contain a smoothing algorithm for the phase interface, whereas the resampled phase interface of ImageJ incorporates fine mesh surface irregularities. For ϕ ≤ 20 vol%, the open *ssa* is smaller than the total *ssa* because the majority of the pores are not connected. For ϕ = 30 vol% most pores are connected and the open *ssa* approaches the total *ssa*. For ϕ = 50 vol%, open and total *ssa* are nearly identical because at this point there are practically no closed pores (see Table 2). Trend lines are plotted defining the mean value of all different calculation methods for total (solid) and open (dashed) *A*_0_ and *ssa*.

## 4. Heat Conduction Modelling

The governing steady-state heat conduction equations within the solid phase and the stagnant fluid phase are given by:

Solid phase:  ∇(*k*_s_∇*T*_s_) = 0 (11)

**Figure 8:**
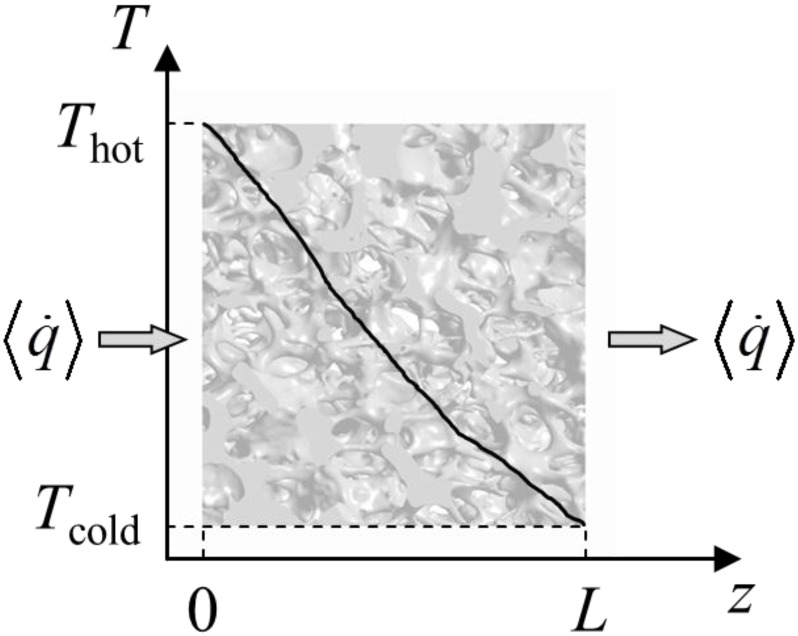
Schematic of the steady state heat conduction simulation setup with hot inlet temperature, *T*_hot_, at *z* = 0 and cold outlet temperature, *T*_cold_, at *z* = *L*.

Fluid phase:  ∇(*k*_f_∇*T*_f_) = 0 (12)

where *k*_s_ and *k*_f_ are the thermal conductivity of the solid and fluid, respectively. The cubic domain is schematically shown in Figure 8. The boundary conditions are given in Equations (13)–(17). An inlet and outlet temperature is set (*T*_hot_ > *T*_cold_) to provide a steady heat flux through the two phases with length *L*. Lateral walls of the sample cube are adiabatic. Local thermal equilibrium is assumed at the phase interface. Heat flux across the interface is driven by the temperature gradient and the thermal conductivities in each phase at the interface.


Inlet temperature:   *T*_s_(*z* = 0) = *T*_f_(*z* = 0) = *T*_hot_ (13)

Outlet temperature:   *T*_s_(*z* = *L*) = *T*_f_(*z* = *L*) = *T*_cold_ (14)

Adiabatic lateral walls:   **n** · 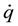 = 0
(15)


Local thermal equilibrium at phase interface: *T*_s_ = *T*_f_ (16)

Heat flux across phase interface:  **n**·*k*_s_∇*T*_s_ = **n**·*k*_f_∇*T*_f_ (17)

The governing volume-averaged steady-state equation for effective heat conduction within the isotropic porous structure reduces to one equation [22,23]:

∇(*k*_eff_∇〈*T*〉) = 0 (18)

**Figure 9:**
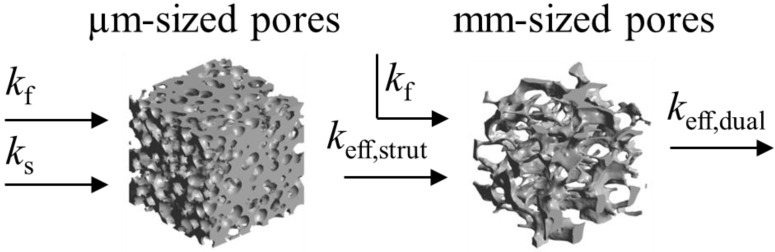
Methodology for the determination of the effective thermal conductivity of the RPC with dual-scale porosity.

The effective thermal conductivity is calculated using the 1D Fourier’s law and the heat flux determined by DPLS:

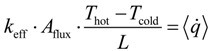
(19)
where *k*_eff_ is the effective thermal conductivity of the cubic porous structure, 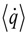 the effective heat flux at the inlet or outlet, and *A*_flux_ = *L*^2^ is the inlet or outlet area constraint with *T*_hot_ or *T*_cold_, respectively. The methodology for determination of *k*_eff_ for dual-scale porous structures is schematically shown in Figure 9. In a first step, *k*_eff_ of the strut with µm-sized pores (*k*_eff,strut_) is determined according Equation (19). In a second step, this *k*_eff,strut_ serves as an input for the solid domain of a further simulation performed with the mm-sized pores of the RPC.

Numerical DPLS are performed for RPC with non-porous/porous struts with/without digital strut dilation for different fluid-solid thermal conductivity ratios ranging from 10^−5^ up to 1. The cases covered include 4 RPC with non-porous struts (original scan and digitally dilated struts with 2, 5, and 10 voxels) and 16 RPC with porous struts (*i.e.*, 4 RPC, each with 4 different strut porosities ϕ* =* 10, 20, 30, 50 vol%). Simulations are performed using a commercial computational fluid dynamics (CFD) software (ANSYS^®^ Academic Research, release 14.0). Initially, grid resolution study is performed, indicating convergence for structures containing element sizes between 0.57 µm at fluid-solid interface to 2.28 µm within bulk for the µm-size pores within the struts, and between 62.0 µm at fluid-solid interface to 247.9 µm within bulk for the mm-size pores of the RPC. Typical number of elements is 20 million, yielding an error of less than 1% compared to the finest mesh tested of 40 million. The correctness of the DPLS was verified by solving simple geometrical cases with exact analytical solutions, while its accuracy was fine-tuned by grid refinement.

**Figure 10:**
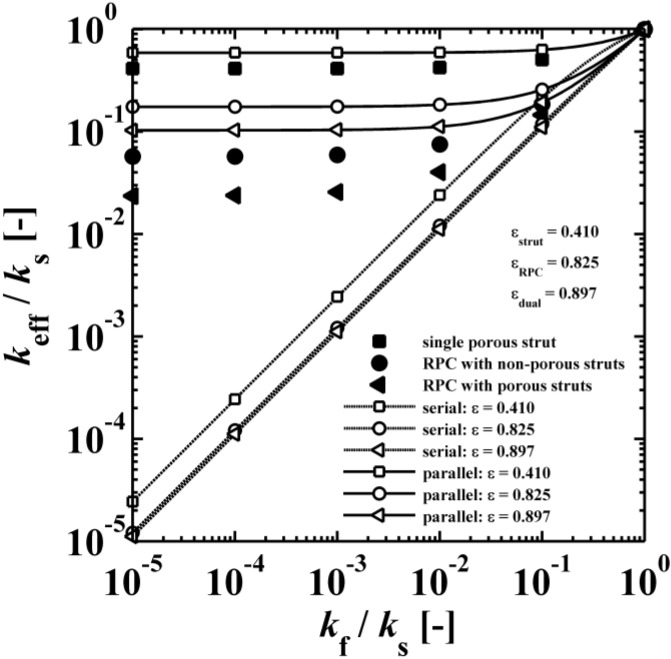
Ratio of the effective thermal conductivity to the solid thermal conductivity* vs.* ratio of the fluid-to-solid thermal conductivity for a single porous strut (ϕ = 50 vol%), a RPC with non-porous struts, and a RPC with porous struts (ϕ = 50 vol%). The analytical curves for serial and parallel slabs are indicating the maximum and minimum possible heat flux.

Figure 10 shows the ratio of the effective thermal conductivity to the solid thermal conductivity *vs.* the ratio of the fluid-to-solid thermal conductivity for a single porous strut (ϕ = 50 vol%), a RPC with non-porous struts, and a RPC with porous struts (ϕ = 50 vol% and 0 mm strut dilation). The analytical curves for serial and parallel slabs are indicating the maximum and minimum possible heat flux [43,44]. These exemplary simulation results correspond to ε_strut_ = 0.410 and ε_RPC_ = 0.825, leading to ε_dual_ = 0.897. *k*_eff_ decreases with increasing porosity and decreasing *k*_f_/*k*_s_. For *k*_f_/*k*_s_ < 10^−3^, *k*_eff_ does not significantly change anymore, indicating heat conduction dominated by the solid domain (e.g., for vacuum applications). In that range, the ratio of *k*_eff_ for RPC with non-porous struts to *k*_eff_ for RPC with porous struts is 2.4. As expected, this ratio approaches 1 for increasing *k*_f_/*k*_s_ as the thermal conductivities of the fluid and solid phases approach each other. The serial and parallel heat conduction mode of the lumped fluid and solid material bracket the minimum and maximum possible heat flux (also called Wiener lower and upper bound), respectively [43,44]. As expected, the simulation results (black symbols) are between the minimum (white symbols with dashed line) and maximum (white symbols with solid line) possible heat flux for each porosity.

Table 4 lists various analytical models for *k*_eff_ [31,45]. For simplicity, analytical equations are given in terms of η = *k*_f_/*k*_s_, and ς_eff_ = *k*_eff_/*k*_s_. Several models allow fitting with geometrical shaping parameters. They were least-squares fitted to three different sets of simulation data: (1) *k*_eff,strut_ for a single porous strut (black squares shown in Figure 10); (2) *k*_eff,RPC_ for a RPC with non-porous struts (black circles shown in Figure 10); and (3) *k*_eff,dual_ for a RPC with porous struts (black triangles shown in Figure 10). To identify the model which agrees best with all data sets, an overall least-squares approximation, *k*_eff,all_, was fitted for all simulation data.

**Table 4:** Analytical models for *k*_eff_.

Model ID	Model	Analytical Expression 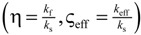	Fitting Parameter
1	Parallel slabs [43,44,46]	ς_eff_ = εη + (1 − ε)	None
2	Serial slabs [43,44,46]	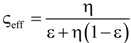	None
3	Hashin and Shtrikman upper bound [47]	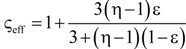	None
4	Hashin and Shtrikman lower bound [47]	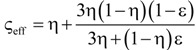	None
5	Woodside & Messmer [48]	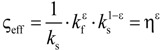	None
6	Russell [49]	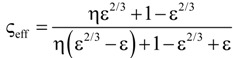	None
7	Loeb [50]	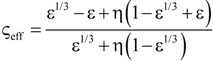	None
8	Maxwell model [45,51,52,53]	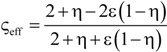	None
9	Schuetz-Glicksmann [54,55]	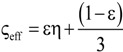	None
10	Bhattacharya * et al.* [56]	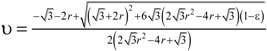	*r*
		χ = 2*r*ν	
		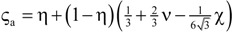	
		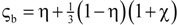	
		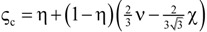	
		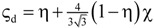	
		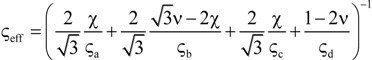	
11	Boomsma and Poulikakos [57]	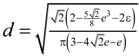	*e*
		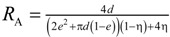	
		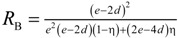	
		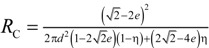	
		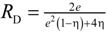	
		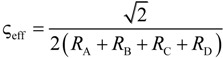	
12	Hamilton [58]	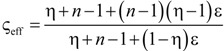	*n*
13	Miller bound [59]	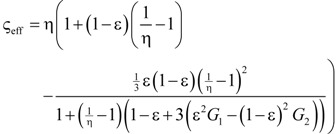	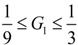
			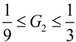
14	Calmidi and Mahajan [60]	ς_eff_ = εη + *A*(1 − ε)^*n*^	*A*
			*n*
15	Dul’nev and Zarichnyak [22,30,61,62]	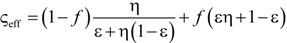	*f*
16	Extended three-resistor model (this work)	*f* = *c*_0_ + *c*_1_ε + *c*_2_ε^2^	*c*_0_
		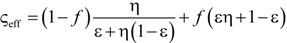	*c*_1_
			*c*_2_
17	Scalable three-resistor model (this work)	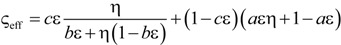	*a*
			*b*
			*c*

**Figure 11:**
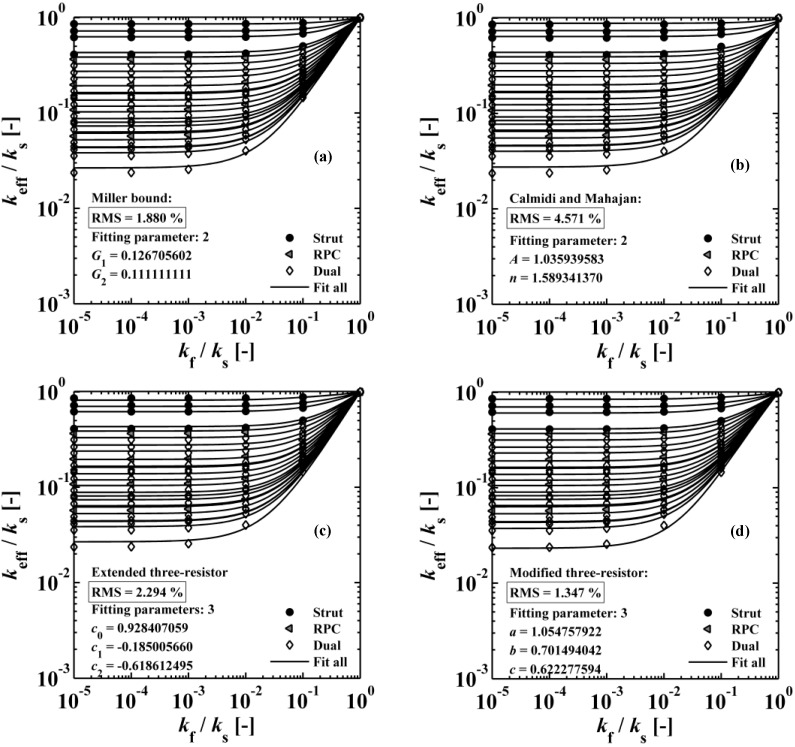
Ratio of the effective thermal conductivity to the solid thermal conductivity* vs.* ratio of the fluid-to-solid thermal conductivity, obtained by our simulation and by least-squares fitted models for structures with a high range of porosities (0.09 < ε < 0.9). Shown are: (**a**) Miller bound model [59]; (**b**) Calmidi and Mahajan model [60]; (**c**) extended three-resistor model; and (**d**) modified three-resistor model. The modified three-resistor model predicts *k*_eff_ with the lowest RMS.

**Figure 12:**
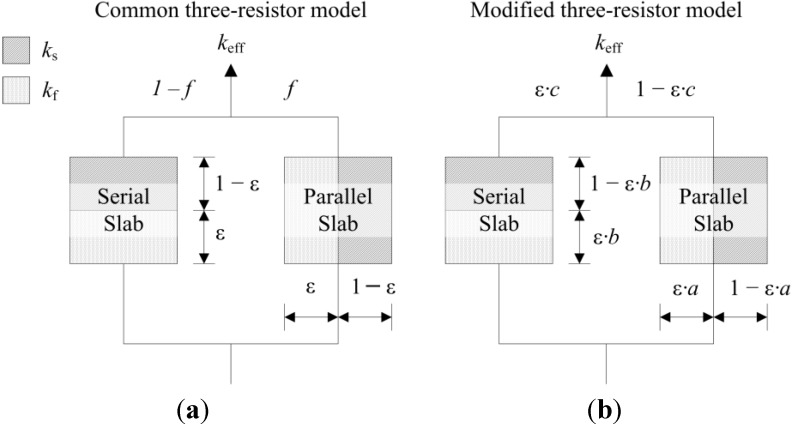
(**a**) Common three-resistor model [22]; (**b**) modified three-resistor model with scaling parameter for the serial and parallel slab and their combination.

The root-mean-square error (RMS) is defined to compare *k*_eff_ calculated by the analytical models with that determined by our simulation:

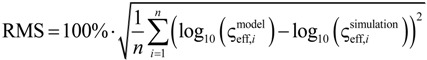
(20)


where *n* is the number of data points per data set over the entire range of *k*_eff_*/k_s_* and *k*_f_/*k*_s_ indicated in Figure 10. Table 5 lists the RMS for the three different simulation data sets and for *k*_eff,all_. Only those models giving an RMS < 5% are considered appropriate. Models 1 to 9, which are not using any geometrical shaping parameter, give RMS > 10%. However, some models perform comparatively well for the prediction of *k*_eff,strut_ with a RMS < 5%: Hashin and Shtrikman upper bound [47] (3.0%), Russell [49] (4.8%), Loeb [50] (2.6%) and Maxwell [51] (3.0%) which is consistent with the findings by Petrasch* et al.* [31] for SiC foams. Serial slab and Hashin and Shtrikman lower bound give very inaccurate predictions of *k*_eff_ (RMS > 200%). This is because the serial bound model assumes no direct connection of solid paths between heat inlet and outlet area, which is obviously not the case for connected, but tortuous strut paths. The Schuetz-Glicksmann model [54,55] yields inaccurate results (21.6%) and predicts values out of the range of the Wiener lower and upper bounds for *k*_f_/*k*_s_ > 0.33. The fitted model of Bhattacharya* et al.* [56] can accurately predict *k*_eff,strut_ (1.5%). However, for the other simulation data sets, RMS > 10%. The model of Boomsma and Poulikakos [57] cannot be applied. Model is not suitable because equations lead to negative length scales in section B (one of four resistances) for any porosity and fitting parameter combination, geometrically describing a non-physical difference between the half node side length and the ligament radius. The fitted model of Hamilton *et al*. [58] gives only *k*_eff,strut_ with RMS < 5%. The fitted Miller’s bound [59] model, shown in Figure 11a, accurately represents the simulation results for *k*_eff,strut_ (0.4%), *k*_eff,RPC_ (1.8%), *k*_eff,dual_ (2.1%) and *k*_eff,all_ (1.9%). This model assumes statistical bound for two-phase media and uses two fitting parameters, *G*_1_ and *G*_2_, including spherical (number 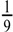) up to platelike (number 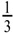) void and solid shapes. Miller’s bound model is restricted within the upper and lower bound of Hashin and Shtrikman [59] for all fitting parameters. The empirical model of Calmidi and Mahajan [60], shown in Figure 11b, is capable of predicting all three data sets and an overall data sets with a RMS < 5%. The model of Dul’nev and Zarichnyak [62] gives only *k*_eff,strut_ with a RMS < 5%. Dul’nev and Zarichnyak [22,30,61,62] propose a model using a linear combination of the Wiener lower and upper bounds with empirical fitting parameter, *f*, for weighting linear combination which is also called three-resistor model. However, if *k*_eff_ is fitted individually for each structure (porosity), an inverse trend of *f* is observed with porosity. Therefore, the three-resistor model is then extended by describing *f* as a 2nd-order polynomial function with porosity. Such extended three-resistor model, shown in Figure 11c, predicts *k*_eff,strut_ with RMS = 0.3% instead of 3.9%, *k*_eff,RPC_ with RMS = 1.6% instead of 9.2%, *k*_eff,dual_ with RMS = 2.2% instead of 9.8%, and *k*_eff,all_ with RMS = 2.3% instead of 12.6%. The three fitting parameters describing *f* with a 2nd-order polynomial function (*c*_0_, *c*_1_, *c*_2_) are replaced to allow the serial and parallel resistance, as well as their combination, to linearly scale with porosity, as shown schematically in Figure 12. Least-squares fitting of this modified three-resistor model, shown in Figure 11d, delivers the most accurate predictions: *k*_eff,strut_ with RMS = 0.1%, *k*_eff,RPC_ with RMS = 1.1%, *k*_eff,dual_ with RMS = 1.4%, and *k*_eff,all_ with RMS = 1.3%. The modified three-resistor model shows the best performance in prediction of *k*_eff_ with overall RMS < 1.5%. Fitting parameter *a* and *b* allow the lumped fluid and solid parts to deviate from actual ε within the parallel and serial slabs, respectively. Fitting parameter *c* allows linear combination of the serial and parallel slab to deviate from ε. This gives some degree of freedom for capturing different tortuous regions for a high porosity range (0.09 < ε < 0.9) and predicts the effective thermal conductivity more accurately compared to linear (or non-linear) combination of parallel/serial bounds and to Miller’s bound model.

**Table 5:** Root-mean-square (RMS) error of analytical models compared to three simulation data sets and to all simulation data.

Model	RMS	*k*_eff,strut_ (*n* = 24)	*k*_eff,RPC_ (*n* = 24)	*k*_eff,dual_ (*n* = 96)	*k*_eff,all_ (*n* = 144)
1	RMS (%)	7.516	26.861	32.158	28.620
2	RMS (%)	233.780	223.485	213.144	218.449
3	RMS (%)	3.010	16.561	20.992	18.466
4	RMS (%)	204.379	207.200	200.080	202.003
5	RMS (%)	63.012	139.992	148.175	136.255
6	RMS (%)	4.772	17.828	22.024	19.497
7	RMS (%)	2.611	15.241	19.916	17.443
8	RMS (%)	3.010	16.561	20.992	18.466
9	RMS (%)	7.516	26.861	32.158	21.621
10	*r*	0.2912	0.1972	0.1254	0.2684
	RMS (%)	1.484	10.738	5.829	13.102
11	RMS (%)	N/A ^1^	N/A ^1^	N/A ^1^	N/A ^1^
12	*n*	2.1985	1.6343	1.5325	1.5701
	RMS (%)	1.247	5.822	6.984	6.868
13	*G*_1_	1/9	0.1262	0.1268	0.1267
	*G*_2_	0.1430	1/9	1/9	1/9
	RMS (%)	0.409	1.828	2.071	1.880
14	*A*	1.0285	1.0482	1.0709	1.0360
	*n*	1.6083	1.5990	1.6095	1.5893
	RMS (%)	2.217	4.721	4.933	4.571
15	*f*	0.8377	0.4954	0.4217	0.4865
	RMS (%)	3.945	9.164	9.754	12.595
16	*c*_0_	0.9972	0.3336	0.2581	0.9284
	*c*_1_	−0.4634	1.6570	1.6780	−0.1850
	*c*_2_	−0.5544	−1.9954	−1.8817	−0.6186
	RMS (%)	0.261	1.611	2.202	2.294
17	*a*	1.3194	1.0823	1.0541	1.0548
	*b*	0.3181	0.6475	0.7008	0.7015
	*c*	0.2000	0.5444	0.6229	0.6223
	RMS (%)	0.135	1.057	1.373	1.347

^1^ Model is not suitable because equations lead to negative length scales in section B (one of four resistances) for any porosity and fitting parameter combination, geometrically describing a non-physical difference between the half node side length and the ligament radius.

## 5. Summary and Conclusions

High and low resolution computer tomographic scans were performed on complex reticulated porous ceramics (RPC) structures to capture the 3D digital representations of their dual-scale porosity in the mm and µm range. The CT scans were processed with a Gaussian blurring filter for a clustering-based image thresholding of the void and solid phases using Otsu’s method. The struts containing µm-size pores were digitally dilated with spherical structuring elements generating structures with different thickness and porosity. The morphological properties analyzed include porosity, pore size distribution, specific surface area, and pore connectivity within representative sample volumes of the isotropic strut regions and of the RPC. The total strut porosity was linearly dependent on the concentration of pore forming agent, and no pore connectivity was observed for concentration less than 20 vol%, consistent with mercury intrusion porosimetry measurements. A well-connected pore network results in high specific surface area and penetration of reactant gas for high fuel production. The effective thermal conductivities of a single porous strut, a RPC with non-porous struts, and a RPC with porous struts (dual scale) were determined by direct pore level simulations of the heat conduction equation with a CFD code. Values were compared to predictions by analytical models over a wide range of porosities. Models without shaping parameters were generally inaccurate (overall RMS > 10%). Miller’s model with two shaping parameters predicted *k*_eff_ with RMS error below 2.1% and the modified three-resistor model with three empirical fitting parameters predicted *k*_eff_ with a RMS error below 1.5%. These analytical correlations are applicable to RPC with porosities in both the strut’s µm-scale and bulk’s mm-scale ranging from 0.09 to 0.9.

The morphological properties and effective thermal conductivity determined in this work serve as an input to volume-averaged models for the design and optimization of solar chemical reactors.

## Nomenclature

*A*_0,(.)_: Volumetric specific surface area (m^−1^)
*A*_flux_: Cross sectional inlet/outlet area of cubic sample for heat flux (m^2^)
*ssa*_(.)_: Physical specific surface area (Index: strut, RPC, dual) (m^2^ g^−1^)
*d*: Diameter of spherical structuring elements (m)
*d*_mean_: Mean pore diameter (m)
*f*(*d*): Pore size distribution (–)
*F*(*d*): Cumulative pore size distribution (–)
*k*_s_: Solid thermal conductivity (W m^−1^ K^−1^)
*k*_f_: Fluid thermal conductivity (W m^−1^ K^−1^)
*k*_eff,(.)_: Effective thermal conductivity of porous structure (W m^−1^ K^−1^)
*l*_REV_: Cube edge length of representative elementary volume (m)
*L*: Cube edge length (m)
*n*: Number of simulation data points (–)
Heat flux through sample (W m^−2 ^)
*s*_2_(*r*): Two point correlation function (–)
*T*_cold_: Cold side temperature (K)
*T*_f_: Fluid temperature (K)
*T*_hot_: Hot side temperature (K)
*T*_s_: Solid temperature (K)
*v*_s_: Voxel size (m)
*V*: Total sample cube volume (m^3^)
*V*_f_: Void volume (m^3^)
ε_(.)_: Porosity (–)
γ: Error band of porosity (–)
ς_eff_: Ratio of effective to solid thermal conductivity (–)
η: Ratio of fluid to solid thermal conductivity (–)
ϕ: Pore former concentration (vol%)

## Subscripts

2pc: 2-point correlation
mesh: 3D-mesh generated from digitally segmented structures
ImageJ: Calculated using open source software ImageJ
strut: Morphological property of porous strut
RPC: Morphological property of RPC with non-porous struts
dual: Morphological property of RPC with porous struts (dual-scale)
open: Open morphological property excluding closed pores
oc: Morphological property after applying opening-closing algorithm
